# IgG and IgG4 antibodies in subjects with irritable bowel syndrome: a case control study in the general population

**DOI:** 10.1186/1471-230X-12-166

**Published:** 2012-11-21

**Authors:** Solveig C Ligaarden, Stian Lydersen, Per G Farup

**Affiliations:** 1 Department of Medicine, Innlandet Hospital Trust, Kyrre Grepps gt 19, 2819, Gjøvik, Norway; 2 Unit for Applied Clinical Research, Department of Cancer Research and Molecular Medicine, Norwegian University of Science and Technology, Trondheim, Norway; 3 Regional Centre for Child and Adolescent Mental Health (RBUP), Department of Neuroscience, Norwegian University of Science and Technology, Trondheim, Norway; 4 Department of Research, Innlandet Hospital Trust, Gjøvik, Norway

**Keywords:** Human, Adult, Irritable bowel syndrome, Cross-sectional studies, Diet, Gastrointestinal tract, Immunoglobulin G, Food Hypersensitivity

## Abstract

**Background:**

Patients with Irritable Bowel Syndrome (IBS) often relate their symptoms to the intake of food and modify their diet. IgE-mediated food allergy is uncommon in IBS, but the role of IgG-mediated food hypersensitivity remains inconclusive. The primary aim of this study was to compare food- and yeast-specific IgG and IgG4 antibodies in subjects with and without IBS.

**Methods:**

This was a case control study in the general population for which subjects completed questionnaires about abdominal complaints and their intake of common food items. Blood samples were collected, and food- and yeast-specific IgG and IgG4 antibodies were measured. Antibodies were measured in mg/L.

**Results:**

We included 269 subjects with IBS and 277 control subjects. After correction for subject characteristics and diet, there were no significant differences with regard to food- and yeast-specific IgG and IgG4 antibodies between subjects with IBS and controls. Lower values of IgG antibodies against egg (OR 0.99 (0.98 to 1.00), p = 0.002) and beef (OR 0.75 (0.60 to 0.94), p = 0.012) and higher values of IgG antibodies against chicken (OR 1.14 (1.03 to 1.27), p = 0.009) were associated with more severe symptoms.

**Conclusions:**

Our findings suggest that IgG-and IgG4-mediated food and yeast hypersensitivity in IBS is unlikely. IgG antibodies against food and yeast may reflect the diet.

## Background

About two-thirds of subjects with irritable bowel syndrome (IBS) relate their symptoms to food intake [1,2]. Most such subjects modify their diet, and a portion of these subjects have an inadequate diet [1]. IgE-mediated food allergy is uncommon and explains the symptoms in only a minority of subjects with IBS [3]. IgG antibodies are purposed to give a more delayed response to specific antigens than IgE antibodies [4], and the subclass IgG4 might induce histamine release, like IgE antibodies, and is found to be synthesized under influence of T-helper 2 cytokines like IgE antibodies [5]. One study found higher titres for some food-specific IgG antibodies in subjects with IBS compared to controls, but no significant correlation between symptom severity and IgG antibody titres [6]. Other studies have shown reduced IBS symptoms when excluding foods for which IgG and IgG4 antibodies were raised [7-9]. The role of IgG-mediated food hypersensitivity is inconclusive [10].

Intestinal colonization of Candida albicans has been described as one cause of IBS symptoms by the popular press [11]. Refined carbohydrates are proposed to facilitate Candida growth in the gastrointestinal tract [12]. One study showed a higher intake of carbohydrates in subjects with IBS compared to controls [13]. The role of Candida albicans in the etiology of IBS remains unclear.

We hypothesised that subjects with IBS would have IgG- and IgG4-mediated food and yeast hypersensitivity. The primary aim of this case–control study was to compare food- and yeast-specific IgG and IgG4 antibodies in subjects with and without IBS in an unselected general population and to relate these values to food intake. Additionally, we wanted to assess associations between the severity of symptoms and levels of IgG and IgG4 antibodies within the IBS population.

## Methods

The OPPHED (Oppland and Hedmark counties) Health Study was conducted in 2000–2001 as a cross-sectional study by the National Health Screening Service (now the Norwegian Institute of Public Health). In this part of the study, all men and women living in Oppland county and born in 1925, 1940, 1955, 1960, or 1970 were invited to participate. Subjects with missing blood samples were excluded, as were all subjects born in 1925 (because of insufficient information).

### Assessments

Subjects were asked to complete questionnaires in paper form. The food and drink questions in our study were the same as the food and drink questions in The Oslo Health Study, which has been translated from Norwegian to English [14]. The following information was gathered from the questionnaires: demographics, smoking habits, activity habits, diet, common diseases (asthma, bronchitis, diabetes, osteoporosis, fibromyalgia, mood disorders, heart attack, angina, cerebral stroke) (number of diseases, score 0–9), allergic rhinitis, mood disorders as measured by the Hopkins Symptom Checklist 10 (HSCL10) (score 1.0-4.0, mental distress ≥ 1.85), musculoskeletal complaints (score 0–12), and gastrointestinal symptoms of which IBS was defined according to a translation of the Rome II criteria [15]. IBS subgroups were classified as constipation-predominant IBS (C-IBS), alternating IBS (A-IBS), or diarrhoea-predominant IBS (D-IBS). Subjects with IBS were matched with controls based on gender and age. The controls were selected out of the dataset as matched cases without IBS. The severity of symptoms (score 1–12) was calculated as the product of severity (mild, moderate, severe (score 1–3)) and frequency (one day or less per week, two to three days per week, four to five days per week, more than five days per week (score 1–4)). Diet was assessed using a limited food frequency questionnaire (FFQ). The questions investigated the frequency and quantity of milk, water, carbonated beverages, and alcoholic beverages, as well as the frequency with which fruits, vegetables, fatty fish, cheese, and omega-3 fatty acid supplements were consumed. The food groups cereals and meat were not included. For the analyses, one dairy product portion was classified as either 150 ml milk/yoghurt or 20 g (one slice) of cheese. Sugar intake was calculated based on the intake of fruits and berries, juice, and carbonated beverages with sugar. Body mass index (BMI) was calculated based on the subject’s measured height and weight. Diastolic and systolic blood pressure and pulse were measured. The IgG antibodies against food panel (FP)5 (milk, egg, cod, wheat, soybean and peanut) and FP73 (beef, chicken, lamb and pork), as well as IgG antibodies against milk, wheat, cod, egg, chicken, beef, pork, brewers’ yeast and *Candida albicans* and IgG4 antibodies against milk, wheat, egg and *Candida albicans* were measured using the ELISA technique (Siemens Healthcare Diagnostics AS). Lower and upper detection limits for IgG antibodies were 2.0 mg/L and 200 mg/L, respectively and for IgG4 antibodies 0.2 mg/L and 50 mg/L, respectively. The upper 95th percentiles of the IgG antibody values as measured against the antigens in the control population were used as reference values.

### Statistical methods

The data were analysed with PASW Statistics 18.0 software (SPSS, Chicago, Illinois, USA). Differences between subjects with and without IBS were assessed using Mann-Whitney’s *U* test, Pearson’s chi-square test, and logistic regression analyses. Variables associated with the severity of symptoms were assessed with ordinal logistic regression analyses. In situations with fewer than 10 observations per covariate, we used backward stepwise elimination terminating in models that included age, gender, HSCL10, and musculoskeletal complaints, and as many other covariates as possible with at least 10 cases per covariate. Sensitivity tests for matched pairs were assessed using conditional logistic regression. Correlations were analysed with Spearman’s correlation. For the regression analyses, missing values were handled by multiple imputations. All variables to be included in the regression analyses were included in the imputation model, which consisted of 62 variables including demographics, dietary intake, common diseases, common blood tests and food- and yeast-specific IgG and IgG4 antibodies. Right skewed variables were log-transformed before use in the imputation model, and 20 datasets were created.

Two-sided p < 0.05 was considered statistically significant. The results are presented as the mean (SD) or odds ratio (OR) with 95% confidence intervals (CI) unless otherwise indicated.

### Ethics

All participants provided their written informed consent before enrolment in the study. The project was approved by the Regional Committees for Medical Research Ethics and the Data Inspectorate, Oslo, Norway.

## Results

**Figure 1:**
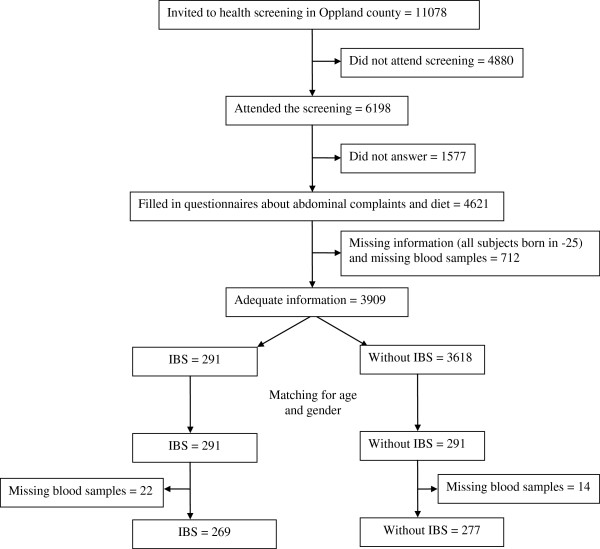
Flow chart.

Of 11078 invited subjects, 4621 completed questionnaires concerning their abdominal complaints and diet (Figure 1). IBS was diagnosed in 291 subjects, and of these subjects, 269 persons were available for inclusion in the study. Constipation predominant IBS was diagnosed in 69, alternating IBS in 120 and diarrhoea predominant IBS in 80 subjects.

**Figure 2:**
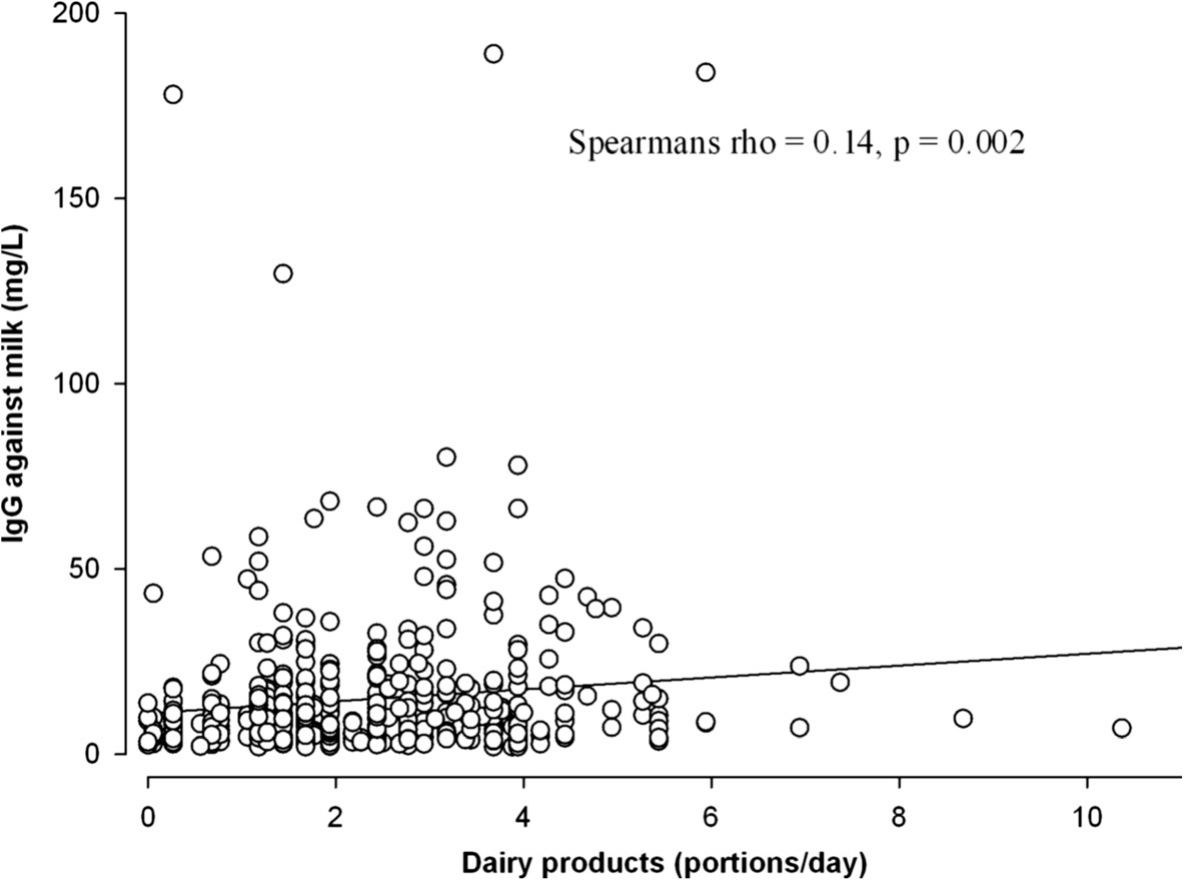
Correlation between intake of dairy products and IgG antibodies against milk.

**Figure 3:**
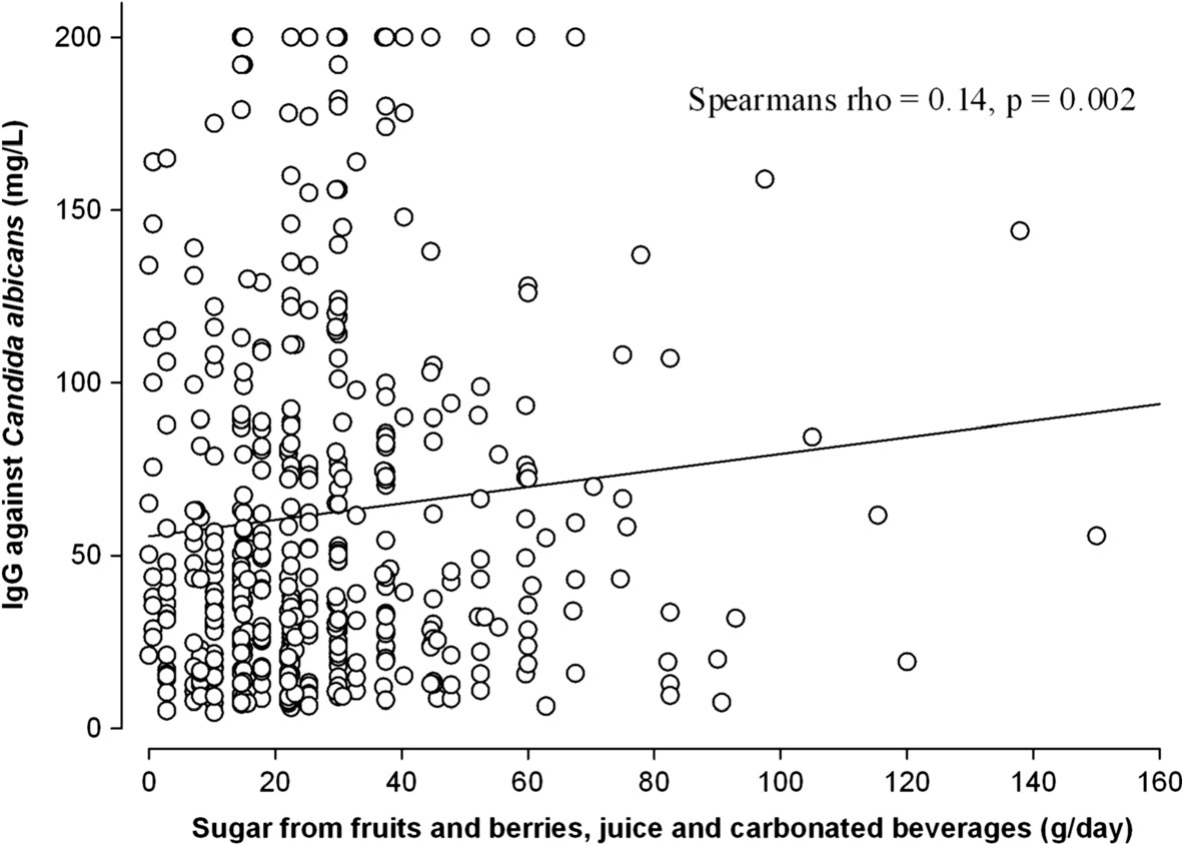
**Correlation between intake of sugar and IgG antibodies against *Candida albicans*.**

Table 1 shows the associations between IBS and subject characteristics, diet, and levels of IgG and IgG4 antibodies against various antigens. Levels of IgG antibodies against milk and wheat were significantly lower among subjects with IBS compared to those without IBS. A lower intake of dairy products was significantly associated with IBS. In the IBS population, the proportion of subjects with IgG antibody levels above the reference values was significantly different for *Candida albicans*: 9.3% (p = 0.035) compared to 5% in the control population. In all subjects, the levels of IgG antibodies against milk were significantly correlated with the intake of dairy products (Figure 2), and the levels of IgG antibodies against *Candida albicans* were significantly correlated with sugar intake (Figure 3).

**Table 1:** Subject characteristics and values for food and yeast antibodies in subjects with and without IBS

***Subject characteristics and food groups***	***IBS***	***Non-IBS***	***p***
	***n = 269***	***n = 277***	
Female, % (0.0)	67.7	68.2	0.89
Age, years (0.0)	43.9 (10.1)	43.5 (10.0)	0.57
BMI, kg/m^2^ (0.2)	26.9 (4.6)	26.6 (4.2)	0.70
Education, years (0.4)	12.0 (3.5)	12.9 (3.3)	0.024
Number of diseases (0–9) (7.5)	0.8 (1.1)	0.5 (0.9)	< 0.001
HSCL10^1^ (1–4) (6.4)	1.6 (0.6)	1.3 (0.4)	< 0.001
Allergic rhinitis, % (4.0)	21.4	10.3	< 0.001
Musculoskeletal complaints (0–12) (34.6)	3.5 (3.0)	2.0 (2.2)	< 0.001
Physical activity, hours/day (10.6)	2.8 (1.7)	3.0 (1.7)	0.11
Smoking, % (1.3)	34.7	35.4	0.87
IgG antibodies FP5^2^ (0.0)	35.7 (35.1)	38.8 (37.0)	0.12
IgG antibodies FP73^3^ (0.0)	6.5 (4.6)	6.8 (5.9)	0.46
IgG antibodies against milk, mg/L (0.0)	13.0 (13.9)	16.1 (22.9)	0.046
IgG antibodies against egg, mg/L (0.0)	31.8 (37.3)	33.3 (36.4)	0.47
IgG antibodies against wheat, mg/L (0.0)	11.5 (10.9)	13.5 (15.8)	0.039
IgG antibodies against pork, mg/L (0.0)	4.2 (2.2)	4.2 (1.9)	0.60
IgG antibodies against beef, mg/L (0.0)	6.3 (1.6)	6.5 (1.6)	0.24
IgG antibodies against chicken, mg/L (0.0)	5.7 (3.8)	6.2 (5.1)	0.091
IgG antibodies against cod, mg/L (0.0)	4.1 (2.8)	4.0 (2.1)	0.55
IgG antibodies against brewers’ yeast, mg/L (0.0)	9.0 (7.3)	8.5 (6.0)	0.40
IgG antibodies against *Candida albicans*, mg/L (0.0)	66.8 (56.1)	55.5 (46.8)	0.057
IgG4 antibodies against milk, mg/L (0.0)	7.9 (13.0)	8.6 (12.9)	0.13
IgG4 antibodies against egg, mg/L (0.0)	12.8 (16.9)	14.4 (17.7)	0.31
IgG4 antibodies against wheat, mg/L (0.0)	1.3 (3.0)	1.8 (5.2)	0.13
IgG4 antibodies against *Candida albicans*, mg/L (0.0)	0.3 (0.4)	0.3 (0.3)	0.62
Fruits and berries, g/day (1.3)	86.6 (76.1)	94.5 (78.9)	0.17
Vegetables, g/day (1.5)	146.9 (118.2)	142.2 (107.1)	0.74
Potatoes, g/day (1.1)	118.6 (78.4)	124.7 (79.8)	0.41
Dairy products, portions/day (12.8)	2.0 (1.5)	2.5 (1.4)	< 0.001
Water, 100 ml/day (1.1)	3.8 (1.9)	3.8 (1.8)	0.78
Carbonated beverages, 100 ml/day (0.0)	2.0 (2.3)	1.5 (1.7)	0.015
Coffee, 100 ml/day (0.4)	4.1 (3.2)	4.6 (3.6)	0.18
Tea, 100 ml/day (0.2)	1.6 (2.6)	1.2 (1.9)	0.22
Juice, 100 ml/day (3.7)	1.0 (1.3)	0.8 (0.9)	0.30
Alcohol, units/day (2.7)	0.24 (0.58)	0.20 (0.29)	0.60
Omega 3 fatty acids, g/day (4.5)	0.7 (0.6)	0.8 (0.7)	0.047
Sugar^4^, g/day (15.0)	30.6 (24.6)	25.5 (16.5)	0.13

^1^Hopkins Symptom Checklist 10 for mood disorder measurement.

^2^food panel with the following allergens: milk, egg, cod, wheat, soybean and peanut.

^3^food panel with the following meat allergens: chicken, beef, pork and lamb.

^4^estimated and calculated from the intake of fruits and berries, juice and carbonated beverages with sugar.

From original data obtained using the Mann–Whitney *U* test/Chi-square test. The ranges for ordinal variables, and the percentage missing, are given in parentheses. Values are mean (SD) unless otherwise.

stated.

In multivariable analyses of associations with IBS, there was a trend toward an association with higher values of IgG antibodies against *Candida albicans* (Table 2). A lower intake of dairy products and higher sugar intake were significantly associated with IBS. There were no significant association between IBS and antibodies against IgG4 or food panels. Analyses without sugar as covariate reduced the p-value of IgG antibodies against *Candida albicans* to 0.035. Analyses without dairy products as covariate reduced the p-value of IgG antibodies against milk to 0.48. Sensitivity analyses with conditional tests and matched pairs yielded similar results.

**Table 2:** Predictors of IBS with values for IgG and IgG4 food antibodies included in the analyses

	**IgG**^**A**^		**IgG**^**B**^		**IgG4**^**C**^	
	**OR (95% CI)**	**p**	**OR (95% CI)**	**p**	**OR (95% CI)**	**p**
HSCL10^1^ (1–4)	3.18 (1.82 to 5.55)	< 0.001	3.32 (1.88 to 5.86)	< 0.001	3.22 (1.84 to 5.62)	< 0.001
Muscle skeletal disease (0–12)	1.14 (1.00 to 1.30)	0.051	1.14 (0.99 to 1.31)	0.063	1.14 (1.00 to 1.30)	0.056
Allergic rhinitis (yes/no)	1.73 (0.99 to 3.02)	0.055	1.72 (0.98 to 3.03)	0.060	1.74 (0.99 to 3.05)	0.053
IgG against FP5^2^, mg/L	1.00 (0.99 to 1.01)	0.83				
IgG against FP73^3^, mg/L	1.00 (0.96 to 1.04)	0.97				
IgG against milk, mg/L			1.00 (0.98 to 1.01)	0.54		
IgG against egg, mg/L			1.00 (1.00 to 1.01)	0.62		
IgG against wheat, mg/L			0.99 (0.97 to 1.01)	0.32		
IgG against cod, mg/L			1.08 (0.97 to 1.20)	0.15		
IgG against pork, mg/L			1.04 (0.91 to 1.20)	0.57		
IgG against chicken, mg/L			0.94 (0.87 to 1.02)	0.15		
IgG against beef, mg/L			0.94 (0.78 to 1.14)	0.54		
IgG against brewers’ yeast, mg/L			1.01 (0.97 to 1.04)	0.65		
IgG against *Candida albicans*, mg/L			1.004 (1.000 to 1.008)	0.051		
IgG4 against milk, mg/L					1.00 (0.98 to 1.02)	0.99
IgG4 against egg, mg/L					0.99 (0.98 to 1.01)	0.43
IgG4 against wheat, mg/L					0.96 (0.91 to 1.02)	0.20
IgG4 against *Candida albicans*, mg/L					1.56 (0.72 to 3.41)	0.26
Dairy products (portions/day)	0.81 (0.68 to 0.96)	0.014	0.80 (0.68 to 0.95)	0.012	0.81 (0.68 to 0.95)	0.011
Sugar (g/day)	1.01 (1.00 to 1.02)	0.043	1.01 (1.00 to 1.02)	0.049	1.01 (1.00 to 1.02)	0.041

All antibodies and other variables with p < 0.1 are shown. Enter logistic regression.

^1^Hopkins Symptom Checklist 10 for mood disorders measurement.

^2^food panel with the following allergens; milk, egg, cod, wheat, soybean and peanut.

^3^food panel with the following meat allergens; chicken, beef, pork and lamb.

Variables included:

A: age, gender, smoking, number of diseases, allergic rhinitis, HSCL10, muscle skeletal disease, IgG against FP5, IgG against FP73, dairy products, vegetables, potatoes, coffee, tea, water, alcohol, omega 3 fatty acids, sugar.

B: age, gender, smoking, number of diseases, allergic rhinitis, HSCL10, muscle skeletal disease, IgG against milk, IgG against egg, IgG against wheat, IgG against cod, IgG against pork, IgG against chicken, IgG against beef, IgG against brewer’s yeast, IgG against *Candida albicans*, dairy products, vegetables, potatoes, coffee, tea, water, alcohol, omega 3 fatty acids, sugar.

C: age, gender, smoking, number of diseases, allergic rhinitis, HSCL10, muscle skeletal disease, IgG4 against milk, IgG4 against egg, IgG4 against wheat, IgG4 against *Candida albicans*, dairy products, vegetables, potatoes, coffee, tea, water, alcohol, omega 3 fatty acids, sugar.

Multivariate regression analyses on IBS subgroups versus subjects without IBS showed significant higher values of IgG antibodies against Candida albicans in diarrhoea predominant and alternating IBS. There were significant lower values of IgG antibodies against egg in diarrhoea predominant IBS. Significant lower values of IgG antibodies against beef and significant higher values against fish were found in constipation predominant IBS. IgG4 antibodies and IgG antibodies against food panels were not associated with subgroups of IBS.

Table 3 shows the associations between the severity of symptoms and IgG antibodies in the IBS population. Lower levels of IgG and IgG4 antibodies against egg, lower levels of IgG antibodies against beef, and higher levels of IgG antibodies against chicken were significantly associated with the severity of symptoms. There was a trend toward higher levels of IgG and IgG4 antibodies against *Candida albicans*.

**Table 3:** Severity of symptoms in subjects with IBS

	**IgG food panels**^**A**^		**IgG**^**B**^		**IgG4**^**C**^	
	**OR (95% CI)**	***P***	**OR (95% CI)**	***P***	**OR (95% CI)**	***P***
HSCL10^1^ (1–4)	1.70 (1.03 to 2.79)	0.038	1.78 (1.07 to 2.95)	0.027	1.78 (1.08 to 2.94)	0.024
IgG against FP5^2^	0.99 (0.98 to 1.00)	0.003				
IgG against FP73^3^	1.05 (1.00 to 1.11)	0.060				
IgG against milk, mg/L			0.99 (0.97 to 1.01)	0.41		
IgG against egg, mg/L			0.99 (0.98 to 1.00)	0.002		
IgG against wheat, mg/L			1.00 (0.97 to 1.03)	0.93		
IgG against cod, mg/L			0.97 (0.85 to 1.11)	0.70		
IgG against pork, mg/L			1.07 (0.92 to 1.25)	0.36		
IgG against chicken, mg/L			1.14 (1.03 to 1.27)	0.009		
IgG against beef, mg/L			0.75 (0.60 to 0.94)	0.012		
IgG against brewer’s yeast, mg/L			1.03 (0.99 to 1.07)	0.14		
IgG against *Candida albicans*, mg/L			1.00 (1.00 to 1.01)	0.072		
IgG4 against milk, mg/L					0.99 (0.96 to 1.01)	0.32
IgG4 against egg, mg/L					0.98 (0.96 to 1.00)	0.014
IgG4 against wheat, mg/L					1.02 (0.92 to 1.12)	0.73
IgG4 against *Candida albicans*, mg/L					2.44 (0.096 to 6.23)	0.062
Vegetables (100 g/day)			1.33 (1.03 to 1.72)	0.031		
Coffee (100 ml/day)	1.07 (0.99 to 1.16)	0.080	1.09 (1.01 to 1.19)	0.031	1.07 (0.99 to 1.17)	0.078
Alcohol (units/day)	1.56 (1.03 to 2.37)	0.038	1.51 (0.99 to 2.29)	0.054	1.54 (1.02 to 2.34)	0.042

All antibodies and other variables with p < 0.1 are shown. Enter ordinal regression.

^1^Hopkins Symptom Checklist 10 for mood disorder measurement.

^2^food panel with the following allergens: milk, egg, cod, wheat, soybean and peanut.

^3^food panel with the following meat allergens: chicken, beef, pork and lamb.

Variables included:

A: age, gender, smoking, number of diseases, allergic rhinitis, HSCL10, musculoskeletal disease, IgG against FP5, IgG against FP73, dairy products, fruits and berries, vegetables, potatoes, carbonated beverages, coffee, tea, water, juice, alcohol, omega-3 fatty acids.

B: age, gender, smoking, number of diseases, allergic rhinitis, HSCL10, musculoskeletal disease, IgG against milk, IgG against egg, IgG against wheat, IgG against cod, IgG against pork, IgG against chicken, IgG against beef, IgG against brewers’ yeast, IgG against *Candida albicans*, dairy products, fruits and berries, vegetables, potatoes, carbonated beverages, coffee, tea, water, juice, alcohol, omega-3 fatty acids.

C: age, gender, smoking, number of diseases, allergic rhinitis, HSCL10, musculoskeletal disease, IgG4 against milk, IgG4 against egg, IgG4 against wheat, IgG4 against *Candida albicans*, dairy products, fruits and berries, vegetables, potatoes, carbonated beverages, coffee, tea, water, juice, alcohol, omega-3 fatty acids.

## Discussion

In this study, there was no significant difference between levels of IgG and IgG4 antibodies in subjects with and without IBS when corrected for the effect of other variables. IBS was significantly associated with a lower intake of dairy products and a higher intake of sugar, and there was a trend toward an association between IgG antibodies against *Candida albicans* and IBS. In the IBS population, there were significant associations between the severity of symptoms and IgG and IgG4 antibodies against egg, IgG antibodies against beef and chicken, and a trend toward an association between the severity of symptoms and IgG and IgG4 antibodies against *Candida albicans*.

IgG antibodies against milk were lower in the IBS compared to the control population when not corrected for the effect of other variables. In addition, there was a positive correlation between the intake of dairy products and levels of IgG antibodies against milk. The intake of dairy products weakened the association of IBS and IgG antibodies against milk. Many subjects with IBS refer to dairy products as trigger foods [16]. Therefore, they may consume smaller amounts of dairy products. Food-specific IgG values may reflect exposure to a specific food [5]. In support of our results, decreased IgE levels were associated with elevated levels of IgG antibodies against milk in individuals who had developed a tolerance to cow’s milk after experiencing an allergy to the same product [17], and subjects drinking milk had higher levels of milk IgG in their sera than did non-milk drinkers in one study [18].

In the IBS population, the IgG pattern associated with symptom severity could reflect an alteration in the individual’s dietary habits when symptoms are severe. Lower values of IgG antibodies against egg and beef and higher values of IgG antibodies against chicken were associated with the severity of symptoms in our study. Egg and beef where reported as offending food items by many subjects with IBS in Scandinavian studies, while chicken seems to be better tolerated, as few individuals with IBS report chicken as an offending food item [1,2]. Subjects with severe IBS symptoms may consume lower quantities of egg and beef and higher quantities of chicken when symptoms are severe and may subsequently achieve lower levels of IgG against egg and beef and higher levels of IgG antibodies against chicken.

We also found higher levels of IgG antibodies against *Candida albicans* in the IBS population; the severity of symptoms was also positively associated with levels of IgG antibodies against *Candida albicans*. Higher values of IgG against *Candida albicans* may simply reflect greater exposure to *Candida albicans*. Although the sugar variable did not represent the total sugar intake, because sugar weakened the association between IBS and levels of IgG antibodies against *Candida albicans*, sugar seems to be a confounder in the context of the *Candida albicans* IBS association. Although not significant, the sugar intake was higher in the IBS population compared to controls. The diet of the IBS population is reported to be high in carbohydrates [13]. This diet may facilitate the growth of *Candida albicans*, but yeast does not necessarily play a role in the symptomatology of IBS.

Some studies have demonstrated an improvement of symptoms in subjects using an elimination diet with low levels of foods that elevate levels of IgG antibodies. Two studies reported elevated levels of food-specific IgG4 antibodies to common food antigens in patients with IBS [8,9]. Subjects who remained on exclusion diets for six months showed a significant improvement in IBS symptoms after 3 and 6 months. There was, however, no control group. Atkinson et al. showed a significant reduction in symptom score in subjects on a diet free of foods against which the subjects had raised IgG antibodies compared to those on a sham diet [7]. However, the study was criticised for including imbalanced groups [19-22].

### Strengths and limitations

The general population-based design of this study reduces the risk for selection bias. The internal validity of the study was increased by the high sample size, which also reduces the risk for a type II error. The response rate was, however, low, which may have induced a selection bias and reduced the external validity. However, a Norwegian study on non-responders found no evidence of major systematic errors [23]. This study had many parallels with our study with regard to time, design, and response rate. The general population design involves the inclusion of subjects with all grades of severity and subjects who either were or were not regularly seeing a doctor for related conditions. The result may be different in subjects with more severe symptoms and in subgroups of IBS as, for example, those seeking medical advice.

In this study, missing values were handled by multiple imputation. Complete case analysis, which is the default in SPSS and other statistical programmes, would have yielded a reduced sample size in the analyses and, more seriously, introduced bias unless data were missing for completely random reasons.

The FFQ was not validated but has been used in several previous publications [24-26]. The FFQ did not include questions regarding cereals, meat, and many sugar-rich products. There are also limitations linked to the dietary assessment methods. In general a FFQ can never catch the complete diet, as there necessarily will be limitations on how many food items the questionnaire can contain [27]. Additionally, as the FFQ is retrospective the method is hampered by recall bias. The subjects with IBS might recall better than subjects without IBS, as subjects with IBS might consider what kind of food intake causes pain/discomfort. Food diaries are more exact, however, in surveys with many participants it is mostly too demanding to use food diaries.

In addition to the antigens tested in this study, there are many other antigens that could be associated with IBS and/or severity of symptoms. The antigens used in our study were selected to cover the most common staple foods which are described as offending to subjects with IBS. Additionally, possible inhalants could have included.

As we in this study found no reason to use serum levels of food- and yeast-specific IgG and IgG4 antibodies to determine food and yeast hypersensitivity, this may not be the best way to determine food and yeast hypersensitivity. There may as an example be other components than proteins of the offending food that causes the hypersensitivity. Or there might be other material than sera where the antibodies can be detected with higher sensitivity, such as fecal samples or mucosal biopsies. In this study there was however no access to fecal samples or mucosal biopsies.

## Conclusion

We found no evidence for the use of IgG or IgG4 antibodies against food and yeast as a method to diagnose food and yeast hypersensitivity in subjects with IBS. IgG antibodies against food and yeast may reflect food intake, and associations between the IgG antibody pattern and the severity of symptoms may reflect a change in the dietary pattern when symptoms are severe.

## Abbreviations

IBS: Irritable Bowel Syndrome; IgG: Immunoglobulin G; IgG4: Immunoglobulin G4; OR: Odds ratio; CI: 95% confidence interval; HSCL10: Hopkins symptom checklist 10; FFQ: Food frequency questionnaire.

## Competing interests

The authors declare that they have no competing interests.

## Authors' contributions

PGF wrote the protocol. SCL, SL, and PGF performed statistical analyses. SCL wrote the paper under the supervision of PGF. All authors have read and approved the final manuscript.

## Pre-publication history

The pre-publication history for this paper can be accessed here:

http://www.biomedcentral.com/1471-230X/12/166/prepub
